# A move in the right direction: Tracking the traceability of British Thoroughbreds outside of racing

**DOI:** 10.1371/journal.pone.0331968

**Published:** 2025-09-19

**Authors:** Jane Michelle Williams, Saranna Jordan, Laura Friend, Emily Kay, Matilda Edmunds, Helena Flynn, Stephen Wensley

**Affiliations:** 1 Equine Department, Hartpury University, Gloucester, United Kingdom; 2 Horse Welfare Board, London, United Kingdom; Universidade Federal de Mato Grosso do Sul, BRAZIL

## Abstract

Horse welfare within/after racing is often questioned by the public. British Racing’s Horse Welfare Board’s “A life well-lived” strategy provides a blueprint for Thoroughbred welfare, advocating accurate lifetime traceability of horses as essential to achieve this. The Census aimed to establish a population density model for British Thoroughbreds, not actively engaged in racing. Equestrians who owned/kept a Thoroughbred were asked to complete the Census between May and December 2023. Frequency analysis identified patterns in passport compliance, knowledge and understanding of current systems, and profiled Thoroughbred demographics: age, use, and history. Records for 8,256 horses were analysed (margin of error: ± 1%, 99% CI); 98% of horses had a passport, but only 64% were in their current owner’s name despite 90% being aware that they should have changed the horse’s registration details. Horses were predominately owned (91%), were geldings (74%), and aged between 5–14 years (63%); Leisure riding, hacking, and unaffiliated competition were the most common activities participated in; no significant differences in registration compliance occurred between activities. The Census provides an accurate representation of British Thoroughbreds not actively involved in racing totalling 33,600 horses, with 80% traceable. The results offer an insight into owner/keeper decision-making with respect to horse registration and Thoroughbred usage after racing. A need to improve current equine traceability systems through digitalisation and simplification was voiced, alongside enhanced communication strategies to showcase why compliance is important. Ongoing accurate records are essential to support education, research, and strategy to safeguard Thoroughbred welfare across their racing and second careers.

## Introduction

Equestrian sports, particularly horse racing, are becoming subject to increasing public scrutiny questioning if traditional training and management practices are ethical and necessary [1]. This has led broader animal welfare advocates to introduce the debate that equestrianism requires a social license to operate (SLO) in the modern era. A social license to operate showcases legitimacy to users and consumers to counteract practices perceived to be detrimental to wellbeing and unethical [2]. Ubiquitous to this debate in horse racing, is the premise that all stakeholders, regardless of their level in the industry, have a duty of care to engage in ethical practices which optimise horse health and welfare [3,4]. Within British horse racing, the Horse Welfare Board’s “*A life well-lived*” strategy provides a blueprint for the welfare of Thoroughbreds bred for racing [5]. Ensuring the industry has the infrastructure to provide lifetime responsibility for Thoroughbreds is a core aim of this strategy; however, to be able to facilitate this premise, accurate traceability of horses across their racing and post-racing careers is essential.

Traceability is a cornerstone of ensuring animals under human care are managed well and is a crucial component of effective animal health and food safety systems [6]. Demographic data for British livestock are generally available due to statutory data monitoring systems that track animal births, movement, and death, however data for the equine population are historically poor [7.^8^]. Accurate data and an effective traceability database for the UK’s equine population is critical for health, welfare, and trade [9]. Accurate records can facilitate tracking of individual horses throughout their lifetime, can indicate ownership and prevent theft, and enable a precise and rapid emergency response to equine health challenges as observed in the UK for equine influenza in 2019 and equine herpes virus in 2021 [6,10]. An effective system should also facilitate enhanced disease surveillance, enforce current food safety legislation, and enable reliable welfare controls, as well as simplifying and enabling bio-secure movements for sport and national/international trade [9]. Being able to accurately track and trace the life of the Thoroughbred beyond racing also offers opportunities to engage and educate the owners and keepers of former racehorses to ensure they are supported to manage the needs of the horses in their care to meet their individual responsibilities and provide all Thoroughbreds with a good life across all stages of their career.

Traceability databases exist for horses; in the UK, the National Equine Database (NED), a collaborative project between the Department for the Environment, Food and Rural Affairs (DEFRA) and the British Equestrian Federation (BEF) was formed in 2006 to collate passport data from all UK equine passport-issuing organisations (PIOs) [11]. This was developed into the Central Equine Database (CED) to manage equine traceability post the EU Equine Passport Regulation in 2017. The database is managed by Equine Register [12] and holds over 1.5 million equine records from across the 81 passport issuing organisations in the UK. Studies have attempted to estimate the size, composition, and geographical spread of the equine population [for example: 11,13] however, the last full equine census in Great Britain occurred in 1934 [13,14].

In France, equine traceability is achieved through the central census database (SIRE), managed by the French Horse and Riding Institute (IFCE) [6,15]. Effective systems often facilitate engagement with wider industry stakeholders such as veterinarians who provide a professional authoritarian voice that can influence concordance levels amongst the end users: the horse owners [15]. However, systems are only as successful as the number of declared equines within them which relies on active engagement from horse owners, who are legally responsible for notifying the CED and SIRE for any changes during a horse’s life (e.g., sale or change of ownership, and death) [6,9].

Ireland has taken a slightly different approach to monitoring equine traceability; the Department of Agriculture, Food and the Marine has coordinated an annual equine census sent to all registered premises of equid owners and keepers (approximately 29,000) to be completed on a single specified night since 2021 [16]. These data aim to meet the needs of EU legislation and capture the location of horses and enable more accurate record keeping informing disease management, help resolve issues related to lost and straying equines and enable evidence-informed decision-making across the Irish equine sector [16].

### Thoroughbred traceability

At birth, Thoroughbred foals are registered with a relevant stud book, for example Wetherby’s in the UK, however, racehorse ownership is often subject to change. For horses actively engaged in racing or training changes in ownership are generally well managed due to regulatory requirements. However, for horses which do not stay in training, or transition to a second career/s at the end of their racing career, potentially with multiple owners, tracking changes in their circumstances is reliant on owner/ keeper compliance to update stud book information. This process involves updating the horse’s passport each time ownership changes and notifying relevant stud books when the horse is deceased. As no regulated national database for tracking equines currently exists in the UK, being unable to track and trace the existing 40,000 Thoroughbreds estimated to have left the racing industry [17] represents a substantial barrier to the racing industry achieving the ambition to provide an overarching responsibility for Thoroughbred welfare from birth to end of life, representing a considerable risk to the industry’s social license.

The Thoroughbred Census project aimed to take a vital first step to ensuring full traceability of Thoroughbreds in the UK by surveying the existing Thoroughbred population using an adapted census methodology to systematically determine and record the number and ownership status of Thoroughbred horses’ resident in the UK between May and December 2023. Secondary aims were to profile the different Thoroughbred communities active across the UK, to determine if differences exist between census and registered passport records and to gain insight into owner/keeper decision making to help inform future Thoroughbred education and welfare initiatives.

## Materials and methods

Population estimates generally rely on the generation of census data to create accurate population density models [18]; therefore, a quantitative census was employed to record details of current UK resident Thoroughbreds [19]. Census data are normally collected for a defined period [19]. However, the logistical challenges associated with surveying equestrian communities precluded this approach, therefore the census was open and active between the 4th of May to 31st of December 2023, representing a period of eight months to optimise data collection.

### Sampling strategy

The aim of a census is to sample every member of a population, however in reality this is difficult to attain. There are an estimated 75,000 Thoroughbreds in the UK, of which approximately 20,000 are in training and 5,000 are at stud, with the details of the remaining 40,000 former racehorses active outside of the racing industry unknown (17]. *A priori* sample size calculations identified a minimum sample size of 385 horses would be representative of the UK Thoroughbred community with a ± 5% error at the 95% confidence interval. This approach is commonly utilised by the World Health Organisation where sampling is limited by a lack of pre-existing data, for example when surveying vaccination uptake in developing nations [20].

People who were responsible for the care of Thoroughbreds in the UK were the target population and were recruited via non-probability sampling methods. A targeted media and communications strategy coordinated by Great British Racing and Hartpury University supported Thoroughbred Census distribution with the aim to raise awareness of the census to different equestrian communities and promote participation. Activity included press announcements, paid and organic social media campaigns, influencers engagement, competitions, as well as physical and advertising presence at key industry events. Participants were recruited online via sharing a link to the census on selected equestrian related social media (Facebook®) groups and pages, through the media strategy, and accessed both online and paper copy Census surveys during attendance at targeted equestrian events such as the London International Horse Show. Direct emails and media posts facilitated by equestrian member organisations and governing bodies such as Retraining of Racehorses (RoR), British Dressage, British Showjumping, British Eventing, British Horse Society and British Riding Clubs. Individuals were encouraged to share the census to other Thoroughbred owners/keepers using a snowball sampling approach. Ethical approval for the study was obtained from the Hartpury University Ethics Committee (Ethics approval number: 2022−137). Respondents were required to confirm their consent within the Census survey; responses that did not provide consent were removed via to analysis.

Racing industry data related to registration and ownership of Thoroughbreds was also collated through personal communication from relevant organisations e.g. Wetherby’s Stud Book and the Thoroughbred Breeders Association to provide a baseline estimate of the UK Thoroughbred population and to ascertain ownership and registration status. Additionally, equestrian member organisations and governing bodies such as British Eventing and RoR were contacted to request current registration data for Thoroughbred horses.

### Census design

The Census was distributed as an online and hard copy questionnaire with 24 questions, divided into three main sections: 1) horse signalment, 2) horse ownership and 3) horse history (S1 Table). Inclusion criteria required participants to be over 18 years of age and to be a current owner or keeper or loan a Thoroughbred racehorse not actively involved in racing. To be eligible to be included in the Census, horses needed to be a full Thoroughbred registered in a racing stud in any country; be resident in the United Kingdom during the census period; be in the owner’s/ keeper’s possession for the census period; and, be living.

The Census collected no personal data on the human participants apart from their surname, however there was the option to include a contact email or telephone number to facilitate future correspondence to gather horse passport and microchip number information if this was not available at the time of completion, or if the participant consented to be contacted for future research or media activities. The online survey was distributed via Qualtrics™ survey software and was live for six months. The draft survey was tested by five experienced equestrian researchers and five equestrian industry representatives to correct any errors before being piloted with the equestrian community at Badminton Horse Trials. Feedback obtained in the pilot informed the final census design.

### Data analysis

Online data were exported from Qualtrics™ to Microsoft Excel™ Version 2010 (Redmond, WA, USA); hard copy data were entered into the same spreadsheet to create a full dataset, totalling 8,567 records. Triangulation of all data records was undertaken to remove duplication in the final dataset prior to analysis. A total of 8,256 horses were recorded in the final dataset and taken forward for analysis.

Frequency analysis identified the demographic characteristics for the Thoroughbreds recorded, the nature of equestrian activities and disciplines respondents engaged with, ownership and passport details. Mean± standard deviation was calculated for horse age. The percentage of respondents who could identify correctly existing passport reporting requirements was also calculated.

Chi square analyses determined if differences occurred in the frequencies of passport registration compliance occurred between disciplines and activities horses participated in [21]. Data were further categorised into horses who engaged in ridden activities, were field companions (non-ridden horse living in the same field as other horses to provide company), participated in equine assisted activities or were a broodmare. Data met non-parametric assumptions, therefore a series of Kruskal-Wallis analyses identified if differences in ownership status, length of ownership, passport location and passport compliance occurred between these groups. For areas where significant differences were found, Mann Whitney U post-hoc tests identified how these areas differed between the disciplines; significance was set at p < 0.05 [21].

### Thematic analysis

Inductive conventional content analysis of open question responses, within the broader framework of McClelland’s Acquired-needs Theory [22] was undertaken utilising tags (‘open-coding’) to create emergent themes (‘focused coding’) using a grounded theory approach [23].

### Accuracy of the census

A sub-sample of census horse records (16%; n = 1,357 horses) were sent to Weatherby’s UK to sensor-check the accuracy of owner/keeper self-reported data: horse was registered in the owner’s name, passport and microchip information was correct and horses’ studbook and age data were accurate. This proportion was chosen as it provided a statistically robust sample while remaining feasible given the constraints of data processing, resource availability, and third-party data access permissions. The subsample size ensured sufficient power to identify discrepancies or trends in data quality, while also aligning with common practice in large-scale survey validation studies, where full cross-validation of all entries is impractical. This approach enabled a meaningful comparison between respondent-reported information and records held by Weatherby’s, enhancing the overall credibility of the dataset without compromising confidentiality or feasibility.

## Results

### Horse demographics

A total of 8,657 horses were registered on the census; after removing corrupted records and duplicates, information for 8,256 horses was taken forward to analysis, which is representative of the UK Thoroughbred population with a margin of error of 99% ± 1%. The completion rate for individual questions was variable; areas which had the lowest completion rates were related to horses’ history prior to their current owner and knowledge of previous owners. Thoroughbred owners/keepers across the UK regions were represented in the census (S7 and S8 Figs).

Most people (90.9%; 5,613/6,174) own their horse, 3.7% (228/6,174) had their Thoroughbred on loan and 5.4% (333/6,174) were the official keeper (carer) for their Thoroughbred. The most common reasons for people to have a horse on loan were that the horse has been retired to them or was on loan (rehomed) from another source such as a welfare organisation. Horses were commonly in the care of keepers if they were a competition horse, with a hunt, being kept on assisted livery or were undertaking a period of retraining before finding a permanent home.

Most Thoroughbreds (62.9%; 3,729/5,951) registered in the census were aged between 5 and 14 years; 6.1% (360/5,951) of horses were aged between 0 and 4 years, with the remaining 31.2% (1,852/5,951) aged 15 years or over (S1 Fig). More Thoroughbreds registered in the census were geldings (73.9%; 4,422/5,982) than mares and fillies (25.8%; 1,542/5,982), with only 0.3% of horses (18/5982) stallions or colts. Approximately half of Thoroughbreds (53.5%; 3,205/5,987) within the census had been in their owner or keeper’s possession for less than 3 years, less (30.2%;1,806/5,987) have cared for their horse between 3 and 9 years, and 16.0% (976/5,987) have had their Thoroughbred for more than 10 years. Most respondents (67.0%; 3495/5294) had owned at least one other Thoroughbred previously (S2 and S3 Figs). Few (14.6%;1,178/8,070) respondents commented on previous owners for their horse (S2 Fig). Most of these Thoroughbreds (48.7%) had been owned by between 1–3 previous people, 38.6% were still with the original owner/ keeper (i.e., no previous owner after the horse’s racing career ended). While only 7.7% of respondents stated they were unaware of this information.

Six key areas were discussed by respondents for why they currently have a Thoroughbred (Table 1).

**Table 1:** Key reasons why respondents own/loan/keep Thoroughbreds.

Project	To bring on (± to sell on) (with specific purpose and leisure and/or competitive goal) To train and compete Price Availability
Personal connection (with horse/ racing/ rehoming centre)	Worked in/ connected to racing – natural to have former racehorse as own horse | wanted to give something back to the horses Individual connection with specific horse: fell in love with/ immediate connection with the horse Prior relationship (ridden/ groomed/ owned/ known)
Friend	To love To have fun with To enjoy riding and interacting with To train and/ or compete (pleasure/ leisure |dressage | eventing | riding club | Retraining of Racehorses (RoR)) Companionship Emotional support
Circumstance/ fate	Serendipity Emotional connection: immediate and established Gifted Reasonable price (compared to other horses) Took on as a ‘*problem*’ horse “*Rescued*” (to give ‘*best life’*| from racing | as abandoned | to prevent being put to sleep) Required companion (horse)
Thoroughbred as a breed	Previously had a Thoroughbred and wanted another: “*everything could want*” Love Thoroughbreds Temperament Athleticism Versatility Talent Kindness
To give something back	To provide good life for horse Because horse deserved opportunity to have a good life, experienced home To give best opportunity after racing New start: To support giving (horse) a new job and life after racing

*Respondents were asked an open question in the Census asking why they owned, loaned or were the keeper of a Thoroughbred. Answers were analysed using inductive conventional content analysis to determine higher and lower order themes from the data.*

Access to former racehorses as an economic option to bring on for their own use or to sell was common:

*“We had looked at the cost of other horses and ex racehorses were more affordable. Rewarding, knowing you are providing a second career to an animal that still has so much to offer.”; “We love projects and producing youngsters and an ex-racehorse allowed us to do that as well as offering an ex-racehorse the chance to continue loving life!”*

The horse-human relationship featured highly in peoples’ decision-making when electing to have a Thoroughbred, as did prior personal relationships with individual horses or the racing industry:

*“Worked in racing for four years, love the breed and nature of the horses. Wanted to give one a loving and knowledgeable home after racing.”; “We wanted to give a retired racehorse a second chapter and new life.”*

The desire to develop a connection with the horse to facilitate a friendly companion or familial relationship was also important:

*“To spoil rotten, provide a home for life and to enjoy!”; “To keep me mentally and physically healthy”; “To ride and enjoy and to have a ‘friend’”; “To retrain and compete on, I just adore my ex-racers, makes my heart happy that I’m giving them a lovely home.”*

Fate also seems to be influential in Thoroughbred owner decision-making, with owners and keepers often identifying serendipitous circumstances which lead them to their current horse:

*“A TB was once a saviour to me. He saved me from a very dark place and time in my life. Sadly, he passed away but now I have to have their presence in my life.”; “Everyone asks, “why another TB?”, for me no other breed comes close in all round ability. Their emotional intelligence is overwhelming and their desire to perform well at a job is phenomenal. The perfect partner to be your best friend and climb the ranks in any sphere and have some fun!”.*

The versatility of the Thoroughbred as a breed, both physically and linked to their temperament, were consistently cited as positive attributes which influenced peoples’ decision to have a former racehorse:

*“Racehorses are amazing athletes and retraining them is a challenge and a pleasure.”; “We always have an ex-racehorse for a riding horse. They are good hacking companions, and we love retraining them.”; “I love the versatility of the TB once you have their heart, they will do anything for you. He’s the best horse I have ever owned.”*

The reasons given by first-time owners of Thoroughbreds focused more specifically on a long-term desire (love) to own a Thoroughbred and/or an immediate emotional connection with their horse, acquiring their horse due to them being available when they were looking or because they were offered or asked to rehome the horse. The key reasons they elected to have a Thoroughbred was to retrain them to have fun or compete with many aiming to event their horses, and generally to have fun with them as a partner.

**Fig 1:**
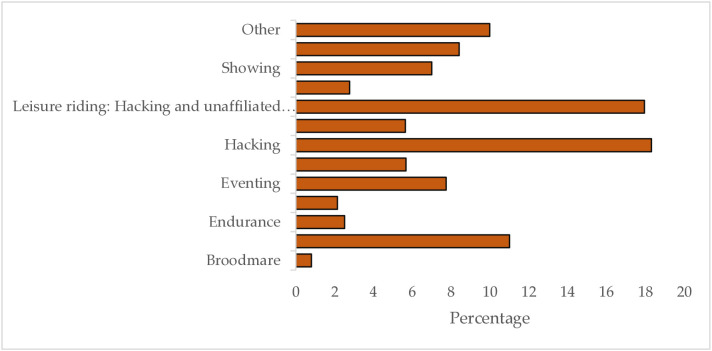
Activities respondents reported undertaking with their Thoroughbreds (n = 8070). Respondents were asked to select which equestrian disciplines and activities they participated in with their Thoroughbred; multiple disciplines could be selected if horses participated in one or more activities listed. The option for ‘other’ could be selected where horses and riders participated in equestrian activities not listed.

The breadth of Thoroughbred communities was reflected in the range of activities it was reported that former racehorses participated in (Fig 1). Hacking, leisure and recreational riding activities, and dressage were the most popular activities and disciplines people indicated they engaged with, with their former racehorse.

Overall, relatively few horses were registered with equestrian member or governing body organisations (S3 Fig); Retraining of Racehorses (RoR) was the most well represented with 36.7% of all horses (2,960/8,069) and 55.4% (980/1,768) of first-time Thoroughbred owners were registered with them. Organisations horses were registered with reflected the activities undertaken with them. Respondents were asked why they did and didn’t engage with RoR, as British Horseracing’s official charity for the welfare of horses who have retired from racing. Four key themes why owners and keepers did register their horse with RoR were identified: 1) Access to training/ clinics; 2) Competitions (showing and RoR classes in particular); 3) Traceability; and 4) Support: education and community. Conversely, six key themes emerged for why owners and keepers stated they had not registered their horse with RoR: 1) Will register when ready to go competing: When going to show; Not doing much at present/ too young; Not sound/ fit enough to warrant yet; 2) Would be happy to register but not sure how to; 3) Not sure what support/ assistance they offer and what the benefits would be; 4) Horse is retired; 5) Lapsed membership; and 6) Not sure if eligible/not eligible as horse didn’t race or was not in training.

Most horses that were registered in the census had previously raced (65,2%; n = 5260) or been in training (64.7%; n = 5224); however, the completion rates for these questions were low suggesting some people completing the census did not know this information for their Thoroughbred. The main reasons for why the horse left racing, reported by their current owner/ keeper, were subdivided into three key themes: 1) Performance; 2) Change in circumstances; and 3) Health (S2 Table).

### Passport/ horse registration details

Most respondents (64%; 3,939/6,157) stated they had changed their horse’s passport, so they are registered in their own name (S4 Fig). It should be noted that approximately a third of respondents (30.2%; 2,500/8,256) did not answer the question related to changing their horse’s passport into their name; removing the 562 people who identified they loaned or were the current keeper of their horse, this leaves 23.5% of Thoroughbred owners who may have not answered due to not having changed their horses passport or not being aware whether they had or not. It appears more common that owners had not changed their passport into their name when they had owned their horse for less than 3 years (54%) or for over 10 years (15%). When asked why they had not updated their horse’s passport details, this was generally due to worries the passport could go missing or a lack of knowledge and understanding of, or frustration with the current process (S3 Table). What owners plan to do with their horse appears to also be influential in decision making, with less owners not identifying a need to change their horse’s registration if they were being used as a leisure horse, hack, or companion.

Most respondents (97.7%; 6,032/6,172) who answered, stated they did have their horse’s passport in their possession; however, across the sample, 26.9% (2,224/8,256) of respondents have not changed their horses’ passport into their name. Top ‘other’ responses for why respondents did not have the horse’s passport were: the passport is with the horse’s current keeper/ loaner (in this circumstance, the current person often has a copy of the passport but not the original), that the horse’s passport is with the breeder, or it is currently in post to update the horse’s registration (S2 Fig). Despite most people having access to their horse’s passport, completion rates were reduced for the questions asking for horses’ passport (59.0%; 4,871/8,256) and microchip (55.4%; 4,576/8,256) details, likely due to issues with access to this information when completing the survey.

**Fig 2:**
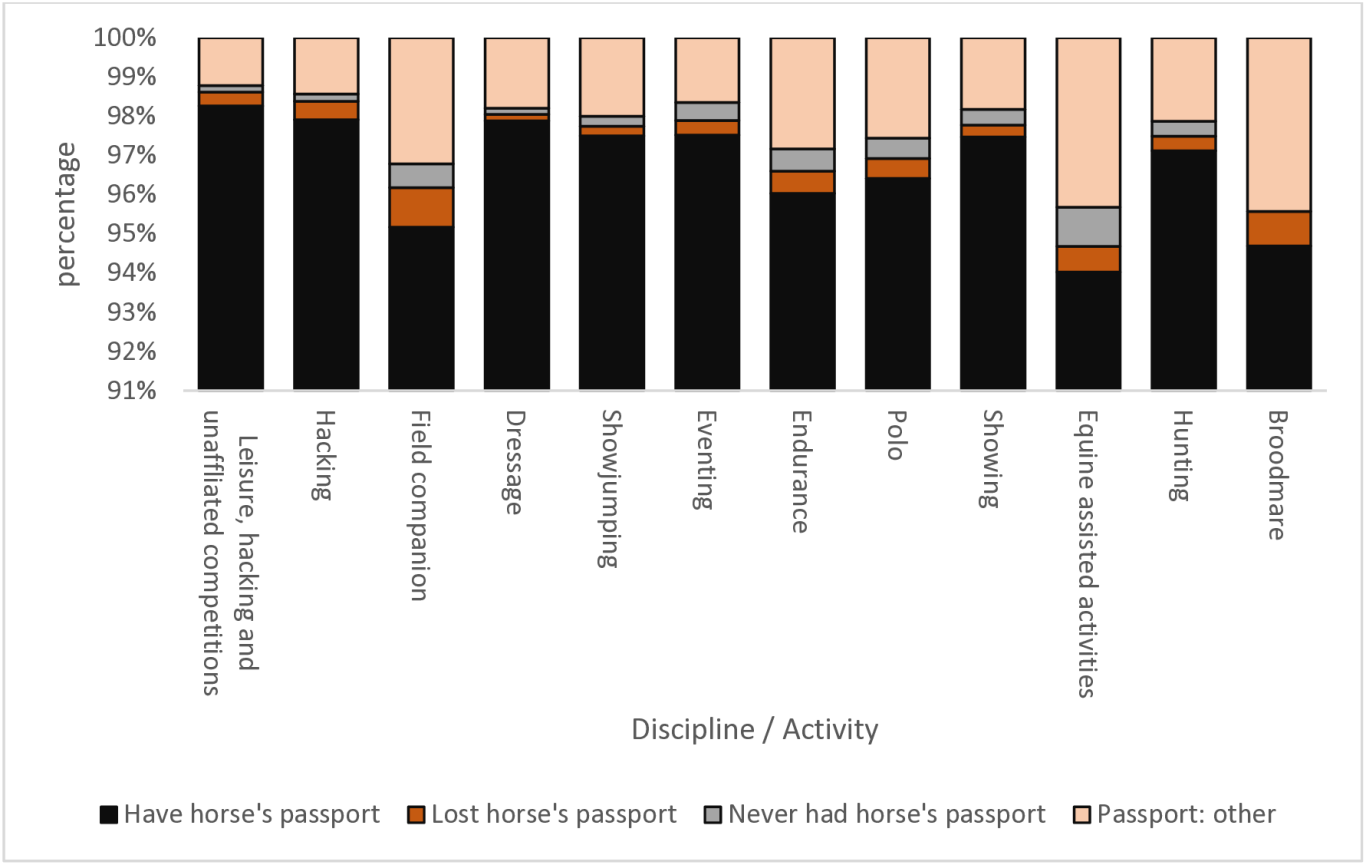
Respondent reported passport status for horses in their care by discipline/ activity. As part of the Census respondents were asked to identify whether they had their horse’s passport in their possession, if they had lost or never had the passport or did not have if for another reason. The percentage of respondents in each passport status category were calculated per discipline or equestrian activity area respondents stated they participated in with their horse.

Chi square analyses identified no differences in the frequency of whether horse owners had registered their horse in their name or not occurred at an individual discipline/activity level (p > 0.05; Figs 2 and S6). However, fewer people who have Thoroughbreds which participate in non-ridden activities such as being a field companion, equine assisted activities and being a broodmare had changed their horse’s passport into their name (S6 Fig). Statistical analysis identified differences existed between the owners/keepers of Thoroughbreds engaged in ridden activities and those whose horses were field companions, participated in equine assisted activities or were broodmares for ownership status (p = 0.03), the length of time horses had been in their owners/keepers care (p = 0.0004), whether they had their horse’s passport (p < 0.001) and if they had changed their horse’s passport into their own name (p < 0.001). Post hoc analyses found former racehorses used for ridden activities are 4.2 times more likely to be owned, 7.3 times less likely to be on loan and 40.2 times less likely to be under the care of a keeper than Thoroughbreds which are field companions (p = 0.027). Thoroughbreds used for ridden activities have been owned for less time than those which are field companions (p = 0.0004), those used for equine assisted activities (p = 0.0004) or those which are broodmares (p = 0.0004); ridden: 57% for less than 3 years compared to 37% for non-ridden activities. Field companions have also been in their owner/keeper’s possession for longer than broodmares (p = 0.007) and horses used for equine assisted activities (p = 0.0004).

Owners and keepers of former racehorses used for ridden activities were 1% more likely to have their horse’s passport in their possession, 50% less likely to have lost their horse’s passport and 37.5 times less likely to have never had their horse’s passport than horses who were field companions (p = 0.009). Owners and keepers of horses used for equine assisted activities were 0.9% less likely to have their horse’s passport, 28.6 times more likely to have lost their horse’s passport and 70 times more likely to have never had their horse’s passport (p = 0.0004) than those of ridden horses. For those owners who had registered their horse into their own name, owners of former racehorses used as broodmares were 4.7 times more likely to have registered the horse in their own name than those whose horses were field companions (p = 0.020). Interestingly, owners and keepers of horses used for equine assisted activities who had registered their Thoroughbred in their own name, were 12.5 times more likely to be registered in the owner’s name than owners and keepers of ridden horses (p = 0.040) and 19.6 times more likely than horses who are field companions (p = 0.006) who has also registered their horses in their name.

Most horses (91.8%; 5,210/5,673) included in the census were registered with Weatherby’s GB (65.8%; 3,733/5,673) and Weatherby’s Ireland (26.0%; 1,475/5,673). Thoroughbreds were registered with other studbooks from around the world reflecting the global racing industry; horses were predominately originally in France (5.1%; 289/5,673), with 3.1% (176/5,673) horses registered in other areas including the USA, Germany, Japan, and New Zealand, with small numbers originating from the Middle East and South America. A small proportion of respondents did not know which studbook their horse was registered with or listed an organisation other than a studbook for where their horse was registered, e.g., British Horse Society. Most people (83.5%; 4,825/5,779) indicated that they would be willing to use a digital or e-passport system to register a change of horse ownership rather than the existing postal system, if this service was free or a nominal fee was charged for using it (S7 Fig).

Most respondents were aware of the requirement to change a passport into their name when a horse’s ownership changed (Aware: 89.9%; Not aware: 10.1%; n = 5,280); however, 20.7% less knew they should also notify the studbook when their horse had died (Aware: 69.2%; Not aware: 30.8%; n = 5,281). Approximately half of respondents were aware they could be fined if they did not register the horse in their name (Aware: 49.7%; Not aware: 50.3%; n = 5,029) but only a quarter of respondents were aware they could be fined if they did not inform the studbook their horse had died (Aware: 24.6%; Not aware: 75.4%; n = 3,617). A substantial proportion of respondents did not attempt the questions relating to horse registration (Ownership: 36.0%, 2,975/8,256; Death: 36.0%: 2,976/8,256) and the potential for fines (Ownership: 39.1%, 3,227/8,256; Death: 56.2%, 4,639/8,256).

### Accuracy of the census

Weatherby’s UK confirmed that 100% of horses sampled were registered with accurate microchip and passport records. Of these 65.4% (n = 888) were registered at Weatherby’s to the owner who completed the census, which represents 7.7% more horses than the reported 26.9% who had not registered their Thoroughbred in their name.

### Traceability: Closing the gap

A key aim of the census was to bridge the gap between the estimated UK Thoroughbred population and reality. Across the last 20 years, an average of 4,900 Thoroughbred foals were born per annum (foal crop in Weatherby’s Fact Book) [24–26], with on average 21,814 horses in training per year (average of BHA horses in training data: 2024−2011) [27], assuming an equivalent number of yearlings to foals are within the industry, this equates to an estimated 31,614 horses actively engaged in the racing industry per year. Retraining of Racehorses estimate that 7,000 Thoroughbreds leave racing per year, of which 40% (n = 2,800) enter retraining for their second careers [28]. Horse age data within the census have identified the average age of former racehorses in the UK is 12 ± 5 years, enabling an informed estimate of the current former racehorse population resident in the UK.

The UK former racehorse population based on the average age of Thoroughbreds owned in the census is therefore: 33,600 horses (lower range: 19,600 horses; upper range: 47,600 horses). Using this population estimate, data collated across the census period can be used to identify the degree of traceability of known Thoroughbreds which have previously raced, been in training or bred for racing within the UK (Table 2). Margin of error calculations based on these figures identify the census results are representative of the UK former racehorse population with a margin of error of ±1% at the 99% confidence level. Therefore, the results presented represent an accurate representation of the UK Thoroughbred population and the behaviour of their owners/keepers and can reliably identify areas where additional research and education initiatives are required to facilitate improved traceability and welfare for former racehorses.

**Table 2:** Estimated UK former racehorse population.

Source	Number of horses* (n = 33,600)
Thoroughbred census	8,256
Retraining of Racehorses (RoR) registered horses	10,205 (12,895−2,690 within the census)
British Showjumping registered TBs	60 ** (149−89 within the census)
Private owners (personal communication outside of census)	53
Point to point registration (22–23 season)	1,632
Polo	1,152 (1,190–38***)
British Dressage	2,982 (3,080 −98****) ^1^
British Eventing (BE)	2579 (2928–265 within the census −84*****)
TOTAL	26,919*†* (80.1%)
Deficit to estimated former racehorse population in the UK	6,681 (19.9%)

*Population estimate based on average age of Thoroughbreds within the Census; **Thoroughbreds registered in British Showjumping RoR classes; ***GB and IRE registered Thoroughbreds between 2000–2024; including reported average annual mortality rate of 3.17% equines (fallen stock) n = 38 [29]; ****: Horses registered with BD with a Weatherby’s GB or IRE or French Galop passport between 2005 and 2024; including average annual mortality rate of 3.17% equines (fallen stock) n = 98 [29]; *****Horses registered with BE with a Weatherby’s GB or IRE passport between 2005–2024; including reported average annual mortality rate of 3.17% equines (fallen stock) n = 84 [29]; †Note: Registered broodmare numbers (n = 7,705) and stallions (n = 116) per annum, are not included in this figure. ^1^NoteBritish Dressage is currently developing its registration system to facilitate breed information in the future.

## Discussion

### Traceability and horse registration

The Thoroughbred Census project aimed to increase knowledge and understanding of the UK Thoroughbred population not actively involved in racing to support the Horse Welfare Board’s strategic aim to improve Thoroughbred traceability to ensure all former racehorses experience a life well lived. The Census has generated intelligence that tracks an estimated 80% of the UK former racehorse population and provides a strong foundation point for ongoing evaluation of future developments to promote horse health and welfare. Most Thoroughbreds (91%) in the UK are owned with a small transient population (~5%) resident with keepers and a further 4% of horses on loan.

A census is an official count and/or survey of a population, often at a particular point in time [30]. Gathering census or fundamental population data at national and local levels is an important first step to underpin the development of policies, planning of services and funding, and enabling an effective response to disasters [8,29]. Globally for livestock species, statutory census-style data monitoring systems that track animal births, movement and death are more widely established than within the equestrian sector [31]. One example is The Cattle Tracing System (CTS)1 run by the British Cattle Movement Service (BCMS) that holds data on the births, movements (linked to sales and breeding), and deaths of all cattle within Great Britain, informing accurate traceability data and providing rich information on the health, performance, and structure of the national cattle herd [31,32]. These existing data provide surveillance intelligence that is widely advocated to inform traceability and can also inform the development of welfare indicators and evaluate risk factors for mortality or improved production values [31–34]. The inaugural Thoroughbred Census provides foundation data that can act as an effective starting point for accurate traceability of the former racehorse herd in the UK that can be built upon in future exercises.

### Passport registration

There was a positive trend that most Thoroughbred owners had changed their horse’s passport into their own name, with no significant differences in registration compliance found across the activities and disciplines horses participated in. Owners who kept their Thoroughbred as a field companion, broodmare or engaged in equine assisted activities were less likely to have their horse’s passport. For broodmares, this is likely to reflect them being at stud and that horses’ passports are correctly in the possession of their keepers; however, for the other two areas, increased education is warranted to showcase the importance of owners having their horse’s passport for example to record vaccination status.

Poor engagement with questions focused on registration, suggest that a wider group of horse owners may have not changed their horse’s registration and did not want to record this behaviour, or perhaps where unaware of why accurate registration records are important. A lack of knowledge and understanding, apathy to complete the process and a lack of recognition for why updating horses’ passports is important were recorded in the Census. In the British Horse Council’s (BHC) 2022 equine identification consultation [9], only 25% of the 3424 horse owners, keepers and equine industry stakeholders surveyed thought equine passport system requirements were communicated well, and respondents reported current processes are complicated, cost too much, are drawn out and difficult. Increased education to showcase the reasons why accurate registration records are important on both an individual (horse/ owner) and population level are needed, including highlighting the collective responsibility all horse owners and keepers have to safeguard the health and welfare of horses in their care. Campaigns to increase knowledge and understanding of the links between traceability, registration and horse health and welfare would be beneficial as part of this process.

### Notification of changes

Most people within the Census were aware of the requirement to record a change in ownership but approximately a third did not realise this should also occur after a horse’s death. The French SIRE database is considered to provide good quality reliable data that informs practice and research [15]. Yet even in this well-respected database, valid contact details for owners and keepers and compliance from horse owners in reporting equine deaths is reported as poor, with the rate of passport return to IFCE just 30–40% and this often occurring a long time after the animal has died precluding accurate traceability records [6,15]. The results of previous equestrian sector consultations [9,35] reinforce that notification of death and sending the paper passport to the PIO is often a sensitive area due to the emotional attachment to the document which can be viewed as a final connection to a much-loved companion. Consideration for how the current system can provide increased reassurance for owners that do not currently engage as they are worried passports will get lost in transit as well as exploring alternative systems that could also meet the needs of horses in loan homes or with transient keepers should be considered in future developments. Management of end-of-life care is an area not best approached for the first time at such a stressful time for the owner [36]. There is increasing debate amongst owners across all animal species to implement quality of life assessments feeding into end-of-life plans for their animals [36–38], perhaps advocating for this approach could have some traction combined with increased education and support to improve compliance but also indirectly support improvements in owner decision-making related to euthanasia. Equine charities such as the British Horse Society and World Horse Welfare may be well placed to support horse owners in this area, but we feel an opportunity exists for RoR to be become a leader providing support for Thoroughbred owners specifically.

### Future development of equine registration

A clear appetite for development of current equine identification systems and increased digitalisation within Thoroughbred registration systems was articulated by Census participants. These results align with the perspectives voiced by horse owners, keepers, and industry stakeholders in DEFRA’s equine identification consultation in 2022 [35], where 65% of owners and keepers strongly or somewhat agreed that records on the CED should be able to be updated digitally to promote timely compliance. Interestingly in contrast, most industry stakeholders (68%) felt a paper passport should be retained for domestic use, e.g., veterinary record keeping and to accompany international movements alongside any digital system.

Whether a hard copy or digital system is utilised, ultimately equine identification systems are only as successful as the quality of data within PIO databases which feed into the CED, and without this quality assurance the value of a central equine database is lost [6.9]. While not asked within this Census, DEFRA’s consultation reported strong agreement (72%) across owners, keepers, and sector stakeholders that the responsibility for notification of changes of ownership and death should be retained by horses’ current and new owners [35]. For UK Thoroughbreds, this relies on active engagement from horse owners to update Weatherby’s regarding changes to ownership and death in a timely manner. Timeframes for mandatory changes in horse registration details were also consulted on by DEFRA, with 66% in favour of a reduced 14-day time limit for changes which would support more timely traceability tracking [35]. Interestingly, two thirds of those surveyed, felt it should be made a legal requirement to record the habitual (home) location of horses on the CED, but only a third agreed that all temporary locations for horses, with a clear definition of what constitutes a temporary domicile needed [35]. Weatherby’s, launched a digital lifetime e-passport for Thoroughbred and non-Thoroughbreds in 2021 [39]. This provides a single, secure platform for all regulatory and legislative identification, movement, health, and welfare requirements, where horse identification, vaccinations and medical records, movement and ownership details can be updated facilitating enhanced and timely equine traceability, that could be a viable solution to expediate registration updates. Across the Census, while respondents were in favour of an increased digital approach, there was a general lack of knowledge and understanding of the e-passport system and communication initiatives to outline the benefits of engaging with this service would be a worthwhile exercise.

However, the challenges to accurate data collection should not be underestimated even where the need for mandatory compliance exists [8,31,40]. Even within established systems such as the CTS challenges in regular data collection exist and it is acknowledged that investment of both time and resources is necessary to move from collecting useful data to generating data that can underpin operational systems [8,31,32]. Challenges can be particularly pertinent, in resource-poor settings, such as the equine sector, were demographic data at national, subnational, and local scales can often be lacking or become quickly outdated due to missing data or inaccurate record keeping [6,8] highlighting how essential accurate traceability is to manage horse health and welfare. Systems which automate data collection, ideally supported by digitalisation, can enable timely access to data which combined with periodic retrospective analysis to ascertain the accuracy of traceability surveillance, are proposed to drive forward research, innovation, and sustainability in livestock farming [8,34]. Therefore, the role of digital technology, further development of the e-passport system, how to effectively police non-compliance, and link with other equine organisations such as RoR and British Dressage [9,35] and how these integrate into future advances in the CED warrants future investigation to drive forward monitoring of Thoroughbred and broader equine traceability to safeguard the UK’s equine population from future disease outbreaks.

### Thoroughbred transition from racing into second careers

As well as playing a key role in public policy decisions and health surveillance, census statistics are acknowledged to contribute to broader social science research [40]. The future of racehorses when they leave the racing industry is one of the primary concerns voiced by the public about the sport [41–43]. Recent equestrian industry reports have highlighted the need to understand the aftercare sector for former racehorses more [5,41,44]. The data from the Thoroughbred Census have increased intelligence on this population, the horses and owners and keepers who took part. Most Thoroughbreds in the Census were aged between 6 and 15 years old; this reflects horses leaving racing after a flat career or those which are not suited to a jump racing career. The age distribution matches the profile for the predominately ridden activities and disciplines their owners and keepers stated they participated in. There is a small but relevant population of aging Thoroughbreds in people’s care with ~15% of horses registered over 19 years of age. Caring for the older horse is often acknowledged as challenging and opportunities to support this group of horses, owners, and keepers to assess and optimise the quality of life of older Thoroughbreds exist [36,37]. Horses registered in the Census were predominantly geldings; this aligns to the dominant male profile of horses in training (geldings: 56.4%; colts: 13% and mares and fillies: 4.3%) [44]. Mares and fillies, due to their sex, may move from racing into studs as their primary second career. The proportion of colts is reduced in the Census as some colts will enter the breeding industry while other horses are likely to be gelded prior to entering retraining. There was a relatively even split across the Census that horses have been owned for either less than or more than 3 years, and that just under half of those surveyed (43%) had previously owned or loaned a Thoroughbred before. Public perception often focuses on wastage and the short duration of racehorses’ careers and lives, and how Thoroughbreds may have multiple homes after they leave racing [41,45]; however, the Census results suggest this is not the case for the majority of former racehorses in the UK and showcase the longevity of Thoroughbred careers post-racing as well as the strong attachment of owners and keepers to the breed.

### Versatility

Thoroughbreds registered in the Census engage in wide and varied activities. Most horses leaving training undergo a period of retraining and/or rehabilitation to educate them to be ridden in non-racing equestrian disciplines and to condition them, physically and mentally to be prepared for these activities [41]. Respondents often stated they purchased their horse with an aim to compete in a specific discipline or to train to be able to do so in the future, to hack and undertaken leisure activities with, or to generally have fun with them. The high percentage of Thoroughbreds recorded in leisure, hacking and unaffiliated competitive homes is pleasing and perhaps reflects how well racing careers can prepare some horses to be suitable for these spheres, in contrast to the commonly held stereotype that all Thoroughbreds are highly strung, with strong flight reactions and not being suitable for these types of careers. A positive human-horse relationship is based on good knowledge of husbandry, skilled handling, care treatment, and an affinity and empathy with animals, dedication, and patience [45]. Within their racing careers, Thoroughbreds often benefit from access to qualified and experienced staff who can positively influence horses’ early education and future relationships through good management and handling practice, and habituating horses with positive loading, clipping etc. as well as benefitting from these skills being developed during retraining [41]. The range of activities and length of ownership reported in the Census supports previous studies in former racehorses that highlight the Thoroughbred’s versatility [42,46].

While it is evident from the Census results that Thoroughbreds can excel in a range of different careers and disciplines, and their owners and keepers feel they develop a strong connection with the people who care for them, not all Thoroughbreds may be suitable for the intention they were purchased for. It was evident that former racehorses are often purchased as ‘project’ horses due to their relatively lower cost to other breeds and their increased availability. This combined with the quite often cited ‘fated’ or ‘immediate’ emotional attachment people stated as influencing their purchase of their Thoroughbred, the largely leisure activities people undertake with them and the lack of knowledge of horses’ prior history, combine into a potential challenge for individual horse-human matches as the level of experience of the human to support the horse’s transition may be lacking, and the expectations of what the horse may progress to do may be unrealistic given their conformation and history [41]. This potential disconnect could negatively impact horse and human wellbeing and stresses the importance of the transition period after a horse’s retirement from racing and the industry being involved in the lifetime care of former racehorses [41,42]. Prior research [46] surveying former racehorse owners suggests the bond between people and their Thoroughbred, often leads to a perception that the horse drives future activities based on what they believe the horse can and cannot physically or psychologically cope with. It is essential for former racehorses to have a good life in their second careers that the needs of the individual horse are met [41–46]. How well the expectations of potential owners and keepers match a horse’s physical and mental capacity should therefore be prioritised [41,46]. Further research is warranted to understand owner and keeper decision-making and how human expectations influence future careers and the lifetime welfare of former racehorses.

### Horse welfare

Horseracing is frequently challenged by the public on welfare and ethical issues related to racehorses during and after racing [42]. The ability to demonstrate that Thoroughbreds have a good life throughout all their life stages including their careers after racing is a strategic priority of global racing jurisdictions [41,42]. Good health and informed traceability are interlinked; how health and human interactions impact on animals’ affective state are fundamental components of the commonly adopted Five Domains Model of Animal Welfare [47–49]. The development of systems to advance positive equine welfare require stakeholders to engage in objective, quantifiable and evidence-informed methods while also adopting a holistic approach to capture the multiple influences that can determine an individual horse’s welfare [49–51]. Accurate demographic knowledge of a population and increased intelligence on how that population interacts with the horses within it, as collated in the Census, not only informs traceability but is also required to assess and model equine health events and contribute to equine welfare strategies [15]. Precise traceability data can therefore provide context for assessment of both herd and individual animal welfare, for example gathering data on the movement of horses from racing into their multiple second careers and how horse owner and keeper influence this demonstrated in the Thoroughbred Census.

### Reasons for Thoroughbreds to leave racing

The most common reasons owners and keepers stated their horse had left racing was due to poor performance and/or a lack of aptitude for the ‘job’. This was often negatively perceived by respondents. Recognition that racing is not the best fit for all Thoroughbreds and that they can progress to second careers should be viewed more positively as this is an example of industry recognition of individual horse’s needs. The next most common reason for horses to leave racing was due to injury and/or health concerns; electing to cease a horse’s racing career in these circumstances could also be viewed as changing their path to support the individual’s needs or conversely, as the industry removing horses which no longer have value, both opinions that were voiced. How the transition process is managed and supported at an individual and sector level will directly influence the future welfare of the horses involved but also shape the public’s perception of how racing is meeting their duty of care to the Thoroughbred and operating within a transparent, credible, and trusted social license [4,41,48].

### Traceability and history

A lack of knowledge and understanding of Thoroughbreds’ prior history was apparent across the Census. This situation is not unique to Thoroughbreds but does represent a partial disconnect to the aspiration of the industry for provision of oversight to ensure the welfare of racehorses from birth to death [5,52]. It also presents challenges for traceability and has the potential to impact negatively on the horse-human relationship between current owners and their horses as they will not know for example the health or injury history of a horse, their normal behaviour and how they have been managed, factors that can influence a successful relationship [4]. An opportunity exists to explore mechanisms for how increased visibility of a former racehorses’ history could be captured and articulated from racing to the second career owners.

### The Thoroughbred-human relationship

Horses and humans have had a long association; relationships take many forms including personal, social, and transactional and they may be positive and/or negative [50]. The strong attachment bonds humans form with their horses and the role this bond has on the success of the horse-human relationship is well documented [53–55]. Developing and maintaining a strong positive horse-human relationship can promote optimal equine welfare, management, and performance and has been shown to have numerous positive physical, social, and psychological benefits for horse owners [55,56]. Conversely the breakdown of this relationship is often associated with deficits in equine management and training systems, can compromise horse and rider safety, and is cited as the reason for equine relinquishment or sales, were these relationships break down [55,56]. A clear pattern emerged in the Census of the strength of the horse: human relationship across the breadth of Thoroughbred communities; owners and keepers consistently identified their ‘love’, passion, commitment, versatility, and attraction to individual horses but also to the Thoroughbred as a breed. This connection was often cited as fated and serendipitous by respondents. The way horse owners and keepers describe their relationship with their horse will influence expectations, attitudes, and behaviours [57,58]. Decision-making informed by an immediate emotional connection, is likely to be subjective rather than apply an objective evidence-informed approach. A subjective approach may not match the horse’s physicality, temperament, and training with the needs (and expectations) of the prospective owner, which could lead to a less-than-optimal future relationship [58–60]

There was also clear evidence from those who are or have previously working in the industry of the bond that racing staff have with the horses in their care, and that they want to give something back and look after them after their racing careers have ended. Racing owners are generally careful and conscientious and frequently take all the necessary steps, often at personal cost, to ensure racehorses move to good homes. The Census findings reflect this, with more than a third of horses (38.6%) still with their original post-racing owner, demonstrating that post-racing owners care for their horses for long periods of time, and racing owners and trainers effectively and carefully select the next step out of racing for their racehorses. Surveys of former racehorse retrainers suggest a similar approach is taken to successfully match horses to suitable owners in their second careers [41]. However, across the sector, further work to understand the complexity of the Thoroughbred-human relationship could enable advancements in welfare, training, husbandry, and management, and help ensure Thoroughbreds experience a good life throughout their career.

### Engagement with the Census

Across the census period, a sense of mistrust regarding why information was being collected and what it would be used for were common themes raised by participants. While the public may appreciate data gathering to support population level surveillance intelligence in a pandemic situation, such as within the Covid-19 pandemic [61], more generic pre-emptive data gathering approaches are often viewed with more trepidation. Managing perceptions regarding data and information gathering through communication strategies that generate a paradigm shift in perception are recommended to showcase how gaining greater knowledge and understanding will help inform improved traceability and tracking of Thoroughbreds throughout their life, enabling this approach to be viewed as a positive sector initiative to support stakeholders to meet collective responsibilities under racing’s social license to operate, and improve registration compliance.

While the Census has been successful and captured a representative view of the former racehorse community in the UK, the results suggest some equestrian communities engage less with racing initiatives aligned to their Thoroughbreds than others. There is potential for racing as an industry to increase engagement and undertake more collaborative initiatives with other disciplines to help engage owners in lifelong traceability and oversight of former racehorse welfare. Recently the British Horseracing Authority, Great British Racing and the Horse Welfare Board have partnered to launch an online hub dedicated to the Thoroughbred: British Racing HorsePWR™: Purpose, Welfare and Responsibility [61,62]. This site aims to engage and educate the public and encourage conversations about racing, the Thoroughbred, their purpose, and the lives they lead, as well as showcasing the high welfare and safety standards that underpin British racing and how the industry are meeting their responsibility to safeguard equine welfare [62].

### Limitations

Census datasets have their respective advantages and limitations. The Thoroughbred Census has captured a single robust dataset representing a ‘line in the sand’ [63] for the UK former racehorse population and provides a spatiotemporal analysis that allows more accurate modelling of census data than assessing than spatial and temporal data alone [64]. However, there are clear gaps in the data captured, which future exercises can target to reduce the potential impact of missing data. Data were self-reported by horses’ owners and keepers and therefore there is a possibility of respondents providing incorrect information or not answering truthfully especially for questions of a sensitive nature or which could be construed by individuals to not be the ‘correct’ or desired response. Low response rates in some question areas suggest that respondents may have strategically elected to refrain from answering questions than indicate a response which could be perceived to be negative. Caution should be applied when interpreting conclusions formed on these data as they may not be representative of the wider population surveyed. However overall engagement with the census has exceeded response rates reported in comparative exercises for the broader equine population in Australia (1.75%) [65] and Ireland (48%) [16].

The Thoroughbred Census is an important keystone moment within the journey to have accurate traceability for Thoroughbreds not actively engaged in racing. While the results have increased our knowledge and understanding of UK Thoroughbreds and factors which influence owner and keeper thought processes related to passport registration, surveys do not allow for a deeper and in-depth understanding of the thought processes which underpin these decisions. Further qualitative studies to delve deeper and gain a greater understanding of individual Thoroughbred communities and how these influence owner and keeper practice and the horses in their care are recommended to help inform future industry education initiatives.

### Recommendations to industry

The inaugural Thoroughbred Census represents the UK Thoroughbred population not actively involved in racing across a point in time; the results identify core areas which can inform Thoroughbred traceability and welfare strategies moving forwards. Collating data such as in the Census, can also help industry to implement welfare improvement interventions and combined with an ongoing commitment to regular monitoring can provide the foundations to appraise the success of welfare improvement activities [28,49–51]. Interestingly, many of the opinions and issues highlighted within the former racehorse population are common to the broader UK equestrian sector [9]; an opportunity exists for the horseracing sector to build on these results and develop existing practice to lead and shape equine traceability in the UK.

To capitalise on the progress made here, we recommend that the results of the Thoroughbred Census should contribute to future strategic planning in racing and across relevant equestrian industry stakeholders. This should include lobbying Government and working with other equestrian stakeholders to further develop equine traceability systems and to move away from the traditional paper passports and legislate a mandatory Digital Equine ID to underpin an efficient and user-friendly system across the UK equestrian sector, to safeguard future equine traceability. Scope also exists to implement education initiatives to improve owner/keeper knowledge and understanding of the links between horse registration, traceability, biosecurity, and welfare and how these relate to being a responsible horse owner. Developing a mechanism to bridge the gap between racing traceability and second careers to ensure 100% of horses can be traced at their first step out of racing, as well as investigating options that could improve information to future owners/keepers of Thoroughbreds to enable them to have details of a horse’s history (health, injury, management, training, temperament) to support transparency and help ensure horse-human matches are suitable for both parties would also be worthwhile. Finally, repeating the Census (or an equivalent exercise) within a 3-to-5-year period to evaluate the impact of the exercise should be undertaken.

## Conclusions

The results represent the first population estimate and overview of UK Thoroughbreds not actively engaged in racing; we estimate that 33,600 Thoroughbreds are resident in the UK and 80.1% of these horses are traceable. Most UK non-racing Thoroughbreds are geldings aged between 5 and 14 years; horses participate in a wide range of equestrian activities and disciplines. Current owners and keepers generally know little about the prior history of their Thoroughbred but feel a strong emotional attachment to them. Approximately two thirds of UK non-racing Thoroughbreds are registered in their owner’s name and most owners/keepers are aware of the requirement to update a horse’s registration when sold but less knew this was also required when a horse died.

The Census provides an in-depth view of the profile of the UK former racehorse community, stakeholder compliance with passport registration and understanding of factors which influence their decision-making. The project has raised the profile of the Thoroughbred and the commitment of British racing to providing lifelong care and ensure traceability of horses after their racing careers, through the extensive reach generated by the media campaign. The results provide a starting point for debate and help identify areas that should be prioritised moving forwards to support Thoroughbred owners and keepers to ensure all Thoroughbreds have a life well lived across all stages of their career.

## Supporting information

S1 Fig Current age profile of Thoroughbreds within the census (n = 5,951). (TIF)

S2 Fig Reported number of previous owners for individual horses (n = 1,178). (TIF)

S3 Fig Respondents (n = 5,294) previous experience of owning, keeping or loaning Thoroughbred former racehorses. (TIF)

S4 Fig Reported passport status for horses registered in the census (n = 8,256). (TIF)

S5 Fig Equestrian organisations Thoroughbreds were registered with (n = 4,753). (TIF)

S6 Fig Percentage of horses registered in their owner’s name by discipline/activity they participate in. (TIF)

S7 Fig Respondent willingness to use an E-passport system to change/update their Thoroughbreds’ passport registration (n = 5,779). (TIF)

S8 Fig Geographical spread of respondents by UK region. (TIF)

S1 Table Overview of question areas asked within the three main sections of the Census. (PDF)

S2 Table Reasons respondents why they have not registered their horse in their name. (PDF)

S3 Table Owner/ keeper reported reasons for why their horse left racing. (PDF)

S1 Video A video summary of the Thoroughbred Census can be downloaded. (DOCX)
