# Supplementary figures and images for: A move in the right direction: Tracking the traceability of British Thoroughbreds outside of racing
Source: PLoS One. 2025 Sep 19;20(9):e0331968. doi: 10.1371/journal.pone.0331968 (PMC12448335; doi:10.1371/journal.pone.0331968)

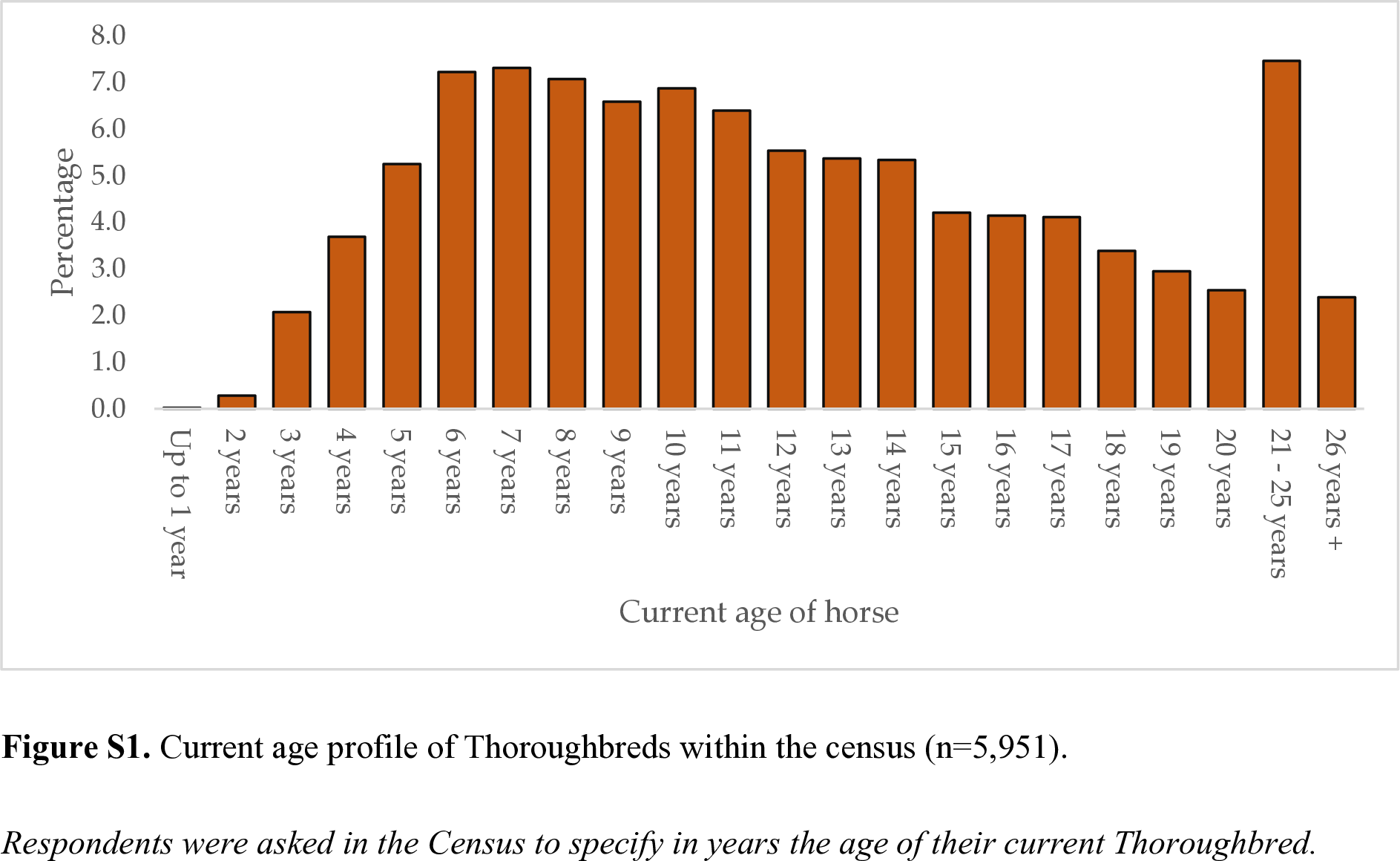

Supplement: S1 Fig — (TIF) [file pone.0331968.s001.tif]

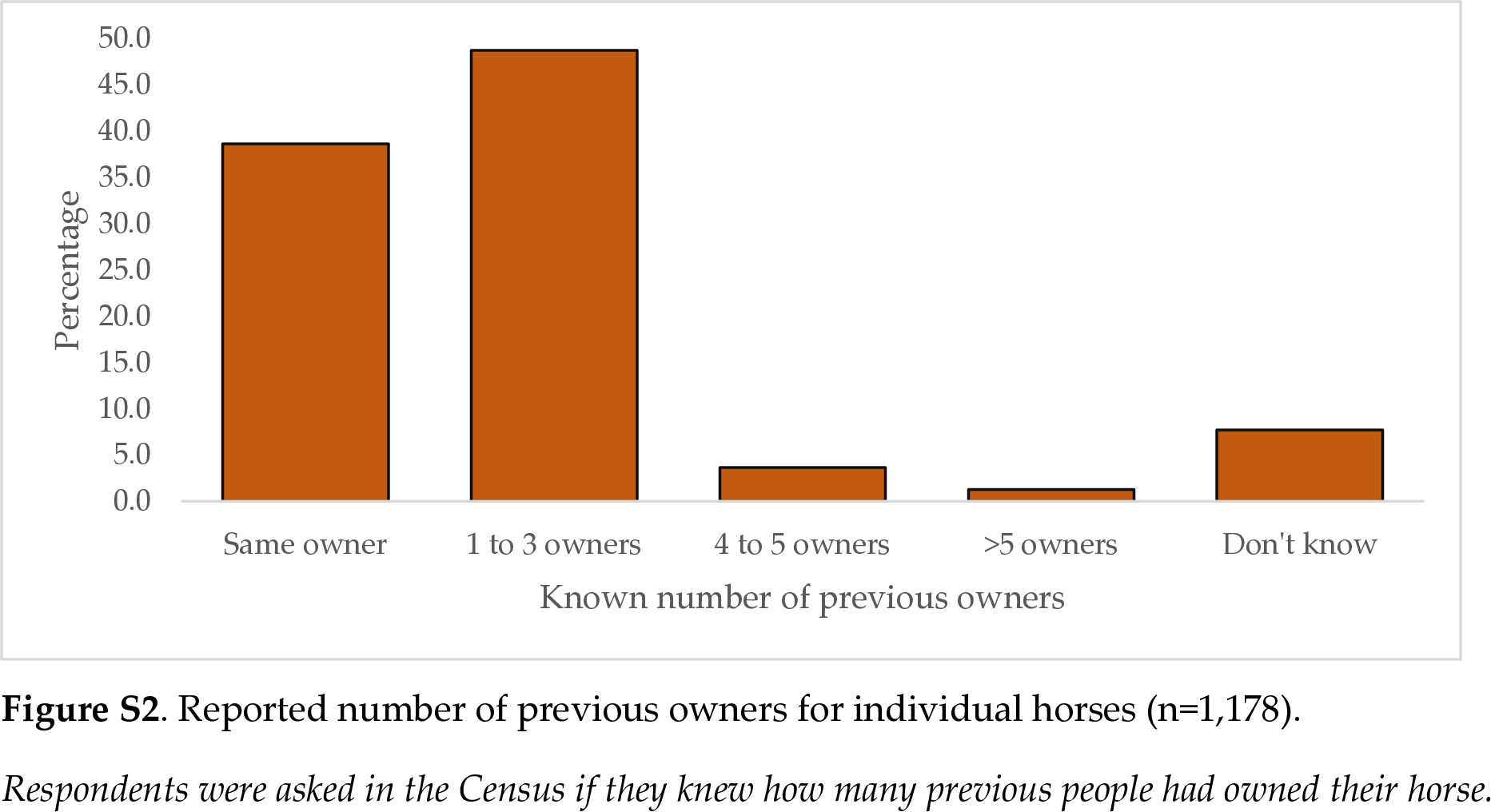

Supplement: S2 Fig — (TIF) [file pone.0331968.s002.tif]

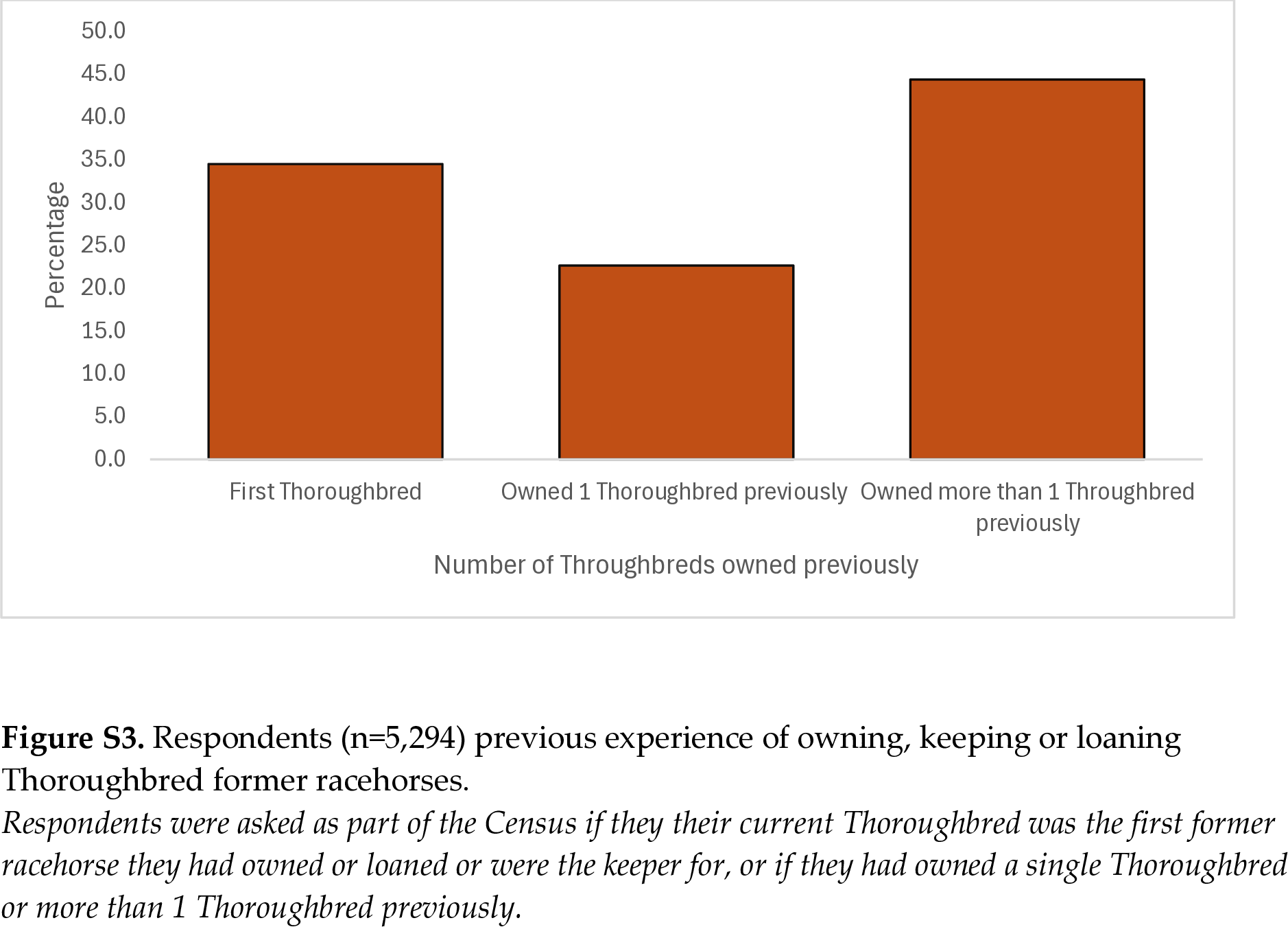

Supplement: S3 Fig — (TIF) [file pone.0331968.s003.tif]

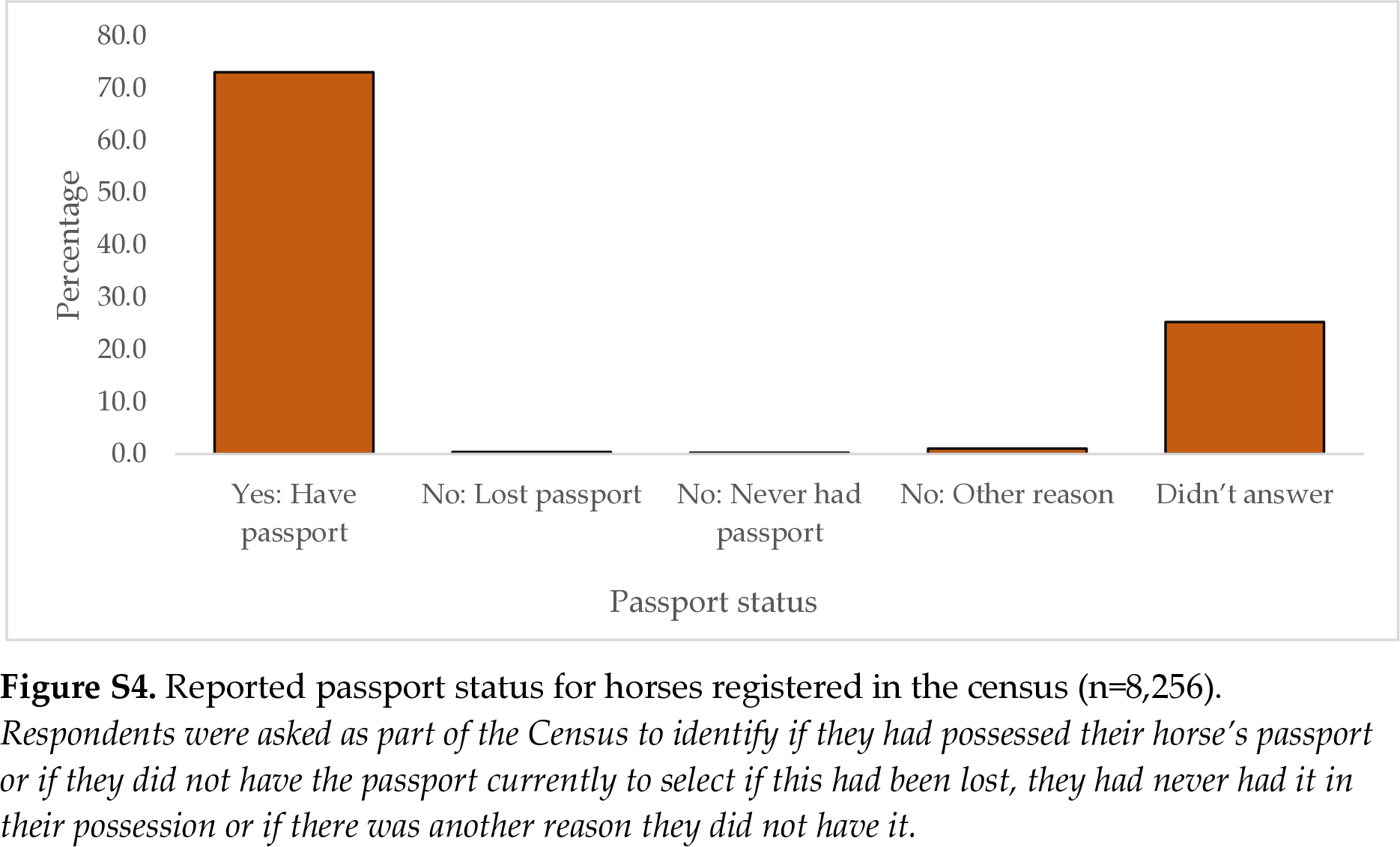

Supplement: S4 Fig — (TIF) [file pone.0331968.s004.tif]

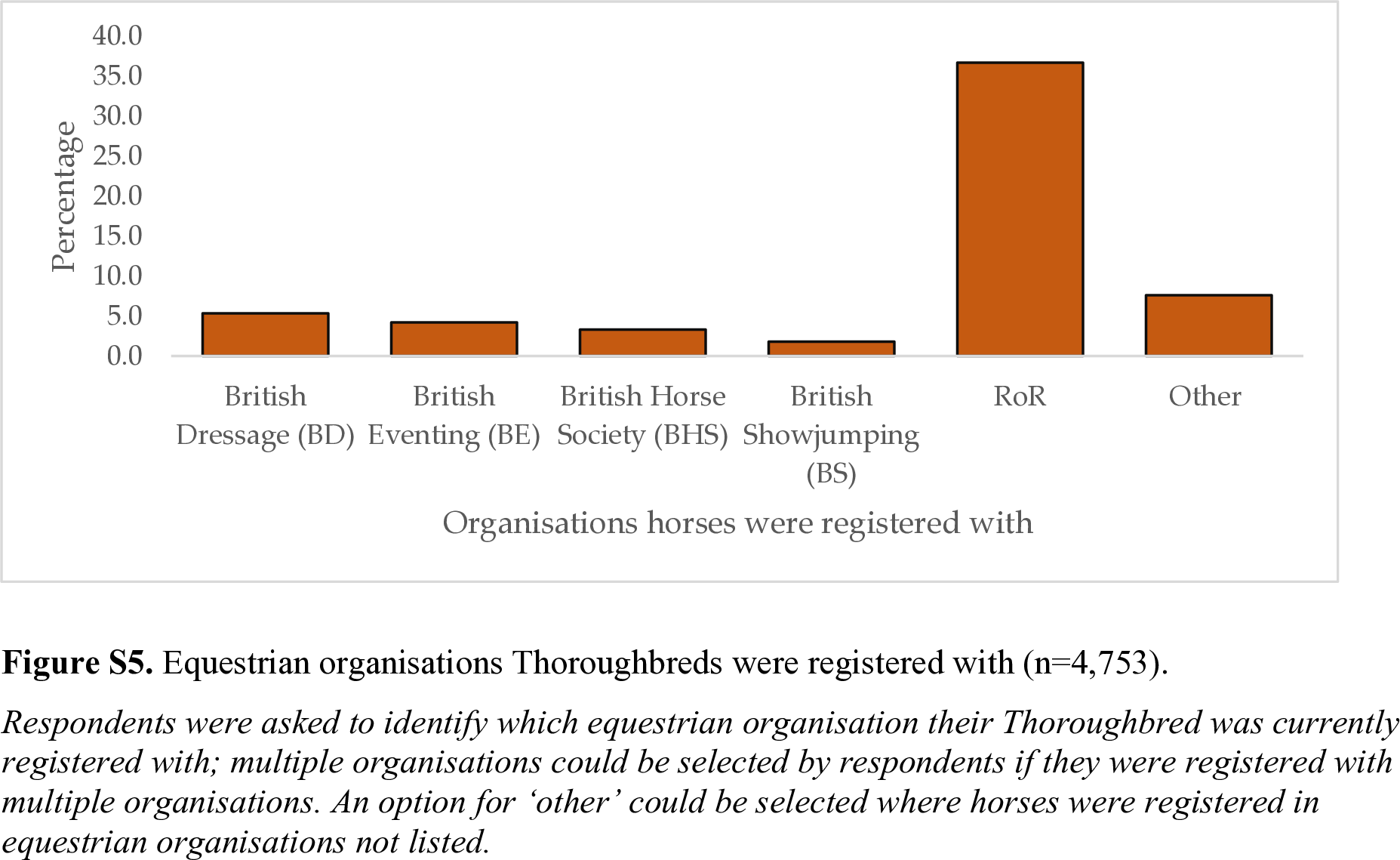

Supplement: S5 Fig — (TIF) [file pone.0331968.s005.tif]

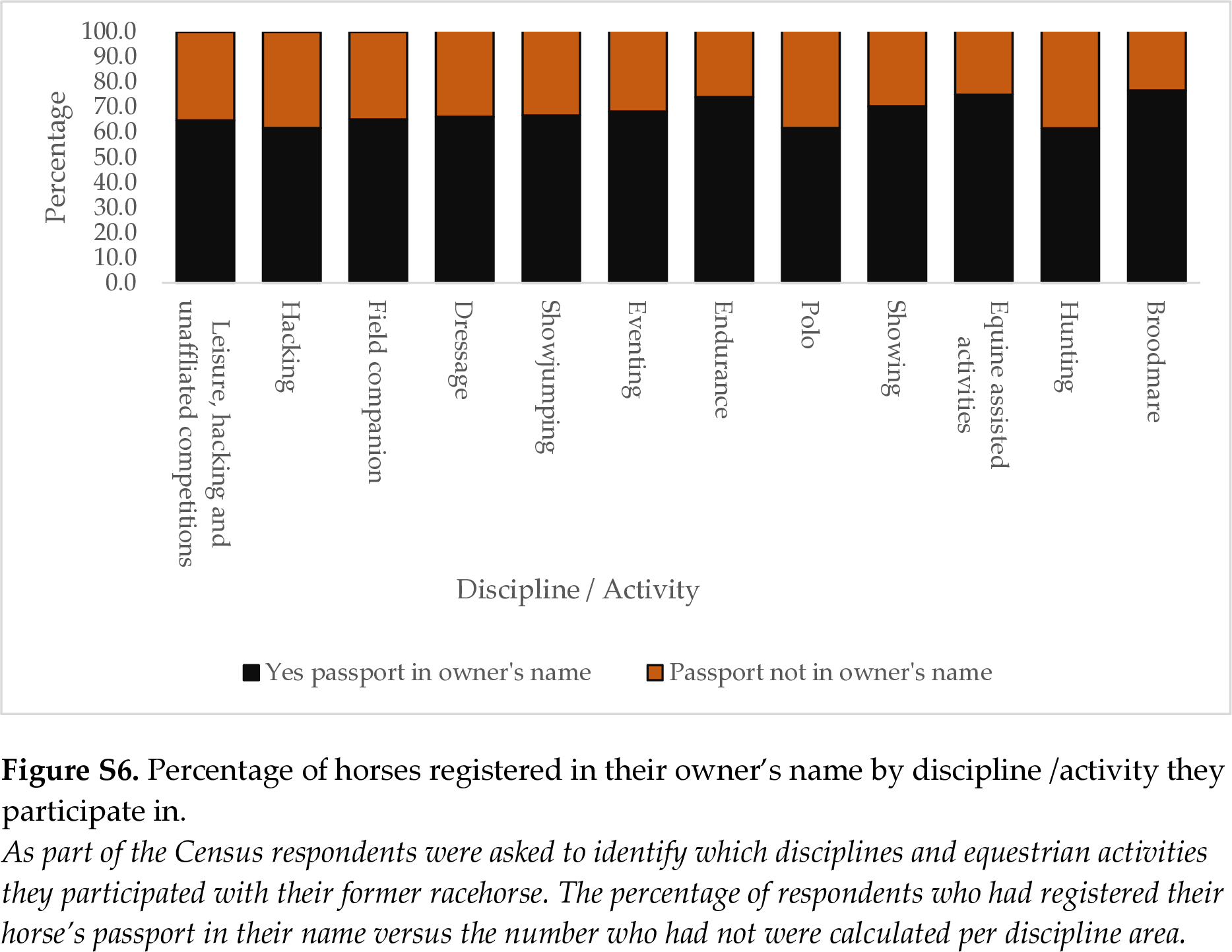

Supplement: S6 Fig — (TIF) [file pone.0331968.s006.tif]

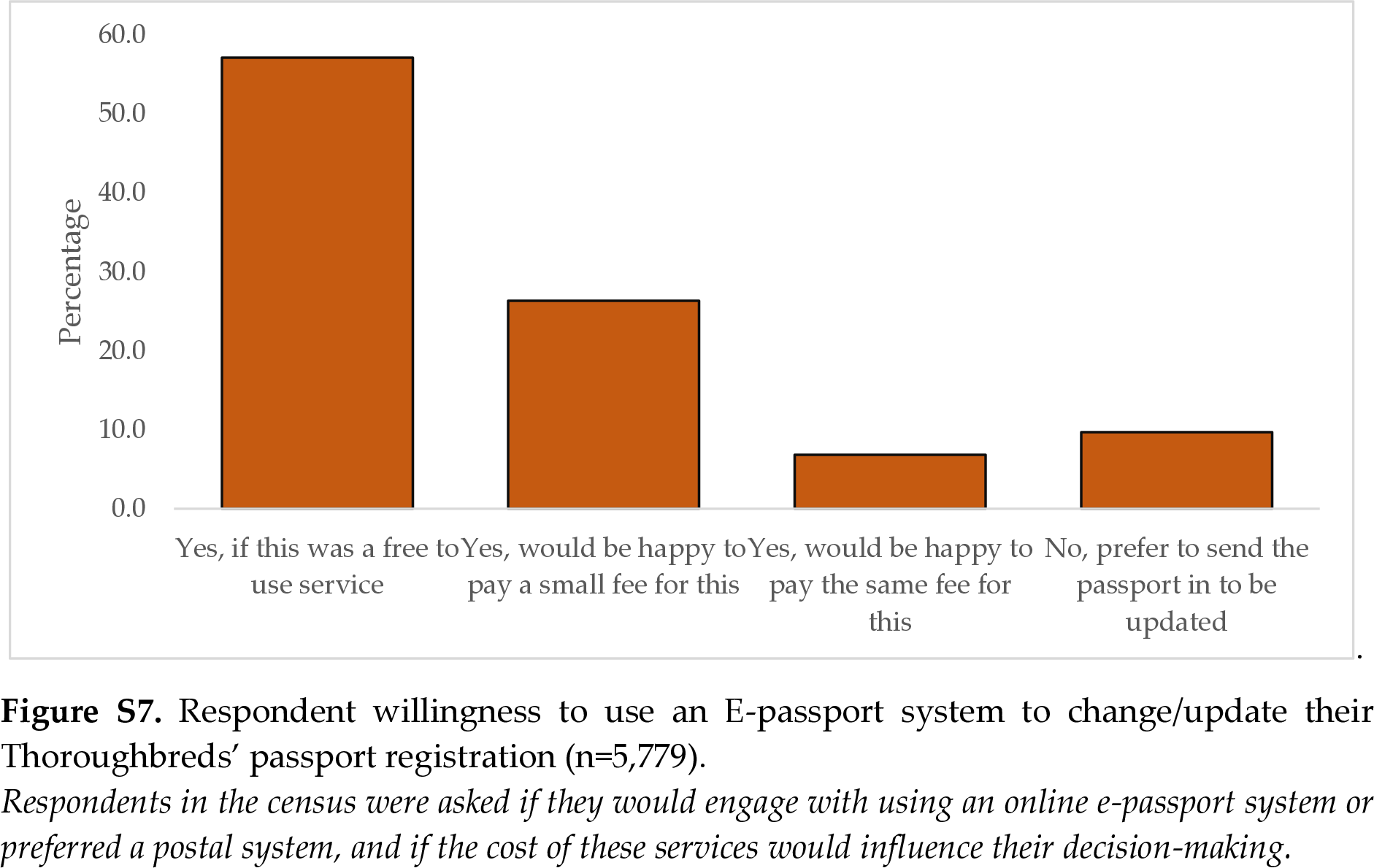

Supplement: S7 Fig — (TIF) [file pone.0331968.s007.tif]

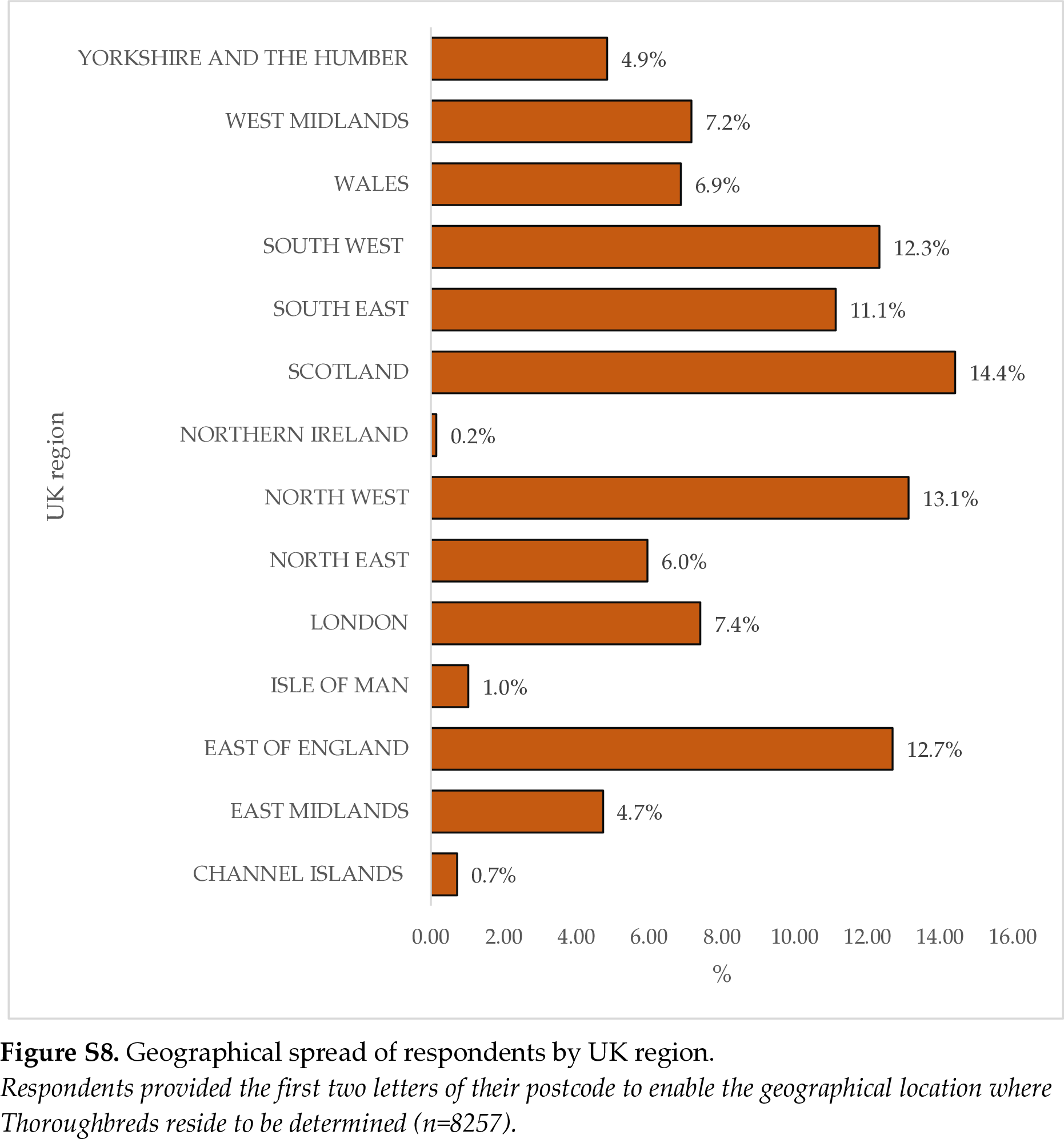

Supplement: S8 Fig — (TIF) [file pone.0331968.s008.tif]
