# Supplementary material for: A move in the right direction: Tracking the traceability of British Thoroughbreds outside of racing
Source: PLoS One. 2025 Sep 19;20(9):e0331968. doi: 10.1371/journal.pone.0331968 (PMC12448335; doi:10.1371/journal.pone.0331968)
Supplement: S1 Table — (PDF) [file pone.0331968.s009.pdf]

British TB Census and racehorse traceability

Supplementary File 1

Table S1. Overview of question areas asked within the three main sections of the Census.

Horse signalment	Stud book horse is registered with
	Passport number
	Microchip number
	Registered name
	Age; Sex; Country of origin
	Have you registered your horse under your name?
Ownership details	Ownership status
	Time with current owner / keeper / loan
	Number of previous owners / keepers
	Resident postcode
	Passport status: registered (human) name
	Location of the passport
	Were you aware you are required to register your horse in your name?
	Were you aware you are required to notify the studbook of your horse's death?

	Were you aware you could be fined for non-compliance with the above?
Horse history	Where was horse sourced /purchased from?<br>Prior racing history<br>Activities undertake with the horse<br>Registrations with equestrian disciplines / member bodies<br>Open questions:<br>Why do you have your horse?<br>Why do you have a Thoroughbred?<br>Reasons for engagement / non-engagement with Retraining of Racehorses<br>Feedback on the census
