# Supplementary material for: A move in the right direction: Tracking the traceability of British Thoroughbreds outside of racing
Source: PLoS One. 2025 Sep 19;20(9):e0331968. doi: 10.1371/journal.pone.0331968 (PMC12448335; doi:10.1371/journal.pone.0331968)
Supplement: S2 Table — (PDF) [file pone.0331968.s010.pdf]

Table S2. Owner / keeper reported reasons for why their horse left racing.

Respondents were asked an open question in the Census asking if they knew the reason/s why their horse left racing. Answers were analysed using inductive conventional content analysis to determine higher and lower order themes from the data.

Performance	• Too slow / not good enough<br>• Poor or reduced racing performance<br>• Lacked aptitude / fell out of love with racing<br>• Lack of race success
Change in horse's circumstances	• Retired<br>• Age (older horses leaving racing due to age only I age associated with reduction in performance / and / or injury)<br>• Change in behaviour (linked to racing / training / soured)<br>• Height: too small<br>• Temperament: too laid back / too highly strung<br>• Financial reasons
Health	• Injury: tendon / ligament / fall<br>• Wind / respiratory issues

<table>
<tr><td>Attachment to horse</td><td>Worried passport will get lost / not be returned
Want to keep (emotional reasons) passport and horse's history
Worried will get lost in the post</td></tr>
<tr><td>Lack of knowledge</td><td>Didn't know how
Didn't know could
Never thought to do this
Nowhere on passport to change name</td></tr>
<tr><td>Circumstance</td><td>On loan
Don't' see worth given horse's use (hack/ leisure / companion): "Won't be doing anything" (competing) with horse therefore don't see need to "he is only a hack"
Transient home (will be moving on to a different home in relatively short timeframe)
Horse is registered in someone else's name:
Family
Partner
Friend
Racing owner
Racing trainer
Charity</td></tr>
<tr><td>Process</td><td>Complexity (especially when horse is registered outside GB)
Poor prior history / experience (passports)
Too much hassle
Remembering to do it
Time (it takes)
Cost
Inconvenient to change name (time):
Competing</td></tr>
</table>

	o Travelling<br>o Under vet treatment<br>o Needed for vaccination record
Procrastination	• Never got round to it<br>• Forgot<br>• Paid for but haven't posted
Expense	• Expensive process<br>• No spare cash (currently)
