# Supplementary material for: A move in the right direction: Tracking the traceability of British Thoroughbreds outside of racing
Source: PLoS One. 2025 Sep 19;20(9):e0331968. doi: 10.1371/journal.pone.0331968 (PMC12448335; doi:10.1371/journal.pone.0331968)
Supplement: S3 Table — (PDF) [file pone.0331968.s011.pdf]

Table S3. Reasons respondents stated for why they have not registered their horse in their name.

Respondents who had not registered their horse in their own name were asked an open question in the Census to explain why this was the case. Answers were analysed using inductive conventional content analysis to determine higher and lower order themes from the data.

Attachment to horse	• Worried passport will get lost / not be returned<br>• Want to keep (emotional reasons) passport and horse's history<br>• Worried will get lost in the post
Lack of knowledge	• Didn't know how<br>• Didn't know could<br>• Never thought to do this<br>• Nowhere on passport to change name
Circumstance	• On loan<br>• Don't' see worth given horse's use (hack/ leisure / companion): "*Won't be doing anything*" (competing) with horse therefore don't see need to "*he is only a hack*"<br>• Transient home (will be moving on to a different home in relatively short timeframe)<br>• Horse is registered in someone else's name:<br>o Family<br>o Partner<br>o Friend

	◦ Racing owner<br>◦ Racing trainer<br>◦ Charity
Process	• Complexity (especially when horse is registered outside GB)<br>• Poor prior history / experience (passports)<br>• Too much hassle<br>• Remembering to do it<br>• Time (it takes)<br>• Cost<br>• Inconvenient to change name (time):<br>◦ Competing<br>◦ Travelling<br>◦ Under vet treatment<br>◦ Needed for vaccination record
Procrastination	• Never got round to it<br>• Forgot<br>• Paid for but haven't posted
Expense	• Expensive process

- No spare cash (currently)
