# Supplementary material for: A move in the right direction: Tracking the traceability of British Thoroughbreds outside of racing
Source: PLoS One. 2025 Sep 19;20(9):e0331968. doi: 10.1371/journal.pone.0331968 (PMC12448335; doi:10.1371/journal.pone.0331968)
Supplement: S1 Video — (DOCX) [file pone.0331968.s012.docx]

**Video Abstract**: A video summary of the Thoroughbred Census can be downloaded at: Video S1: <https://pure.hartpury.ac.uk/files/70158732/British_racing_s_Thoroughbred_Census_animation_-_the_results_are_in_1.mp4>.
